# The Initial Dispersal and Spread of an Intentional Invader at Three Spatial Scales

**DOI:** 10.1371/journal.pone.0062407

**Published:** 2013-05-06

**Authors:** Nadiah P. Kristensen, Paul J. De Barro, Nancy A. Schellhorn

**Affiliations:** Commonwealth Scientific and Industrial Research Organisation, Ecosystem Sciences, Brisbane, Queensland, Australia; University College London, United Kingdom

## Abstract

The way an invasion progresses through space is a theme of interest common to invasion ecology and biological pest control. Models and mark-release studies of arthropods have been used extensively to extend and inform invasion processes of establishment and spread. However, the extremely common single-scale approach of monitoring initial spread leads to misinterpretation of rate and mode. Using the intentional release of a novel biological control agent (a parasitic hymenoptera, *Eretmocerus hayati* Zolnerowich & Rose (Hymenoptera: Aphelinidae), we studied its initial dispersal and spread at three different spatial scales, the local scale (tens of metres), field scale (hundreds of metres) and landscape scale (kilometres) around the release point. We fit models to each observed spread pattern at each spatial scale. We show that *E. hayati* exhibits stratified dispersal; moving further, faster and by a different mechanism than would have been concluded with a single local-scale post-release sampling design. In fact, interpretation of each scale independent of other scales gave three different models of dispersal, and three different impressions of the dominant dispersal mechanisms. Our findings demonstrate that using a single-scale approach may lead to quite erroneous conclusions, hence the necessity of using a multiple-scale hierarchical sampling design for inferring spread and the dominant dispersal mechanism of either human intended or unintended invasions.

## Introduction

Invasion is a multi-step process comprised of three phases: initial dispersal (where an organism moves from its native habitat, often over long distances, to a new habitat outside of its home range); establishment of self-sustaining populations within the new habitat; and spread of the organism to nearby habitats [1]-[3]. Biological control introductions are staged invasions where proliferation is managed by mass rearing and planned release with the hope of initial dispersal, establishment, and spread, and subsequent suppression of a target pest.

Both human intended and unintended biological invasions have movement at the core of their success. Although, the terms movement and dispersal are often used interchangeably, and depending on the discipline can differ, here ’movement’ means a change in the spatial location of an individual [4] and ’dispersal’ means population redistribution that leads to spatial spread of organisms [5]. The term spread, is related, and is most often discussed in the context of non-indigenous organisms expanding their range. Much is known about the spread of arthropods [3], [6]. For example, spread often occurs by stratified dispersal – the combined short- and long-distance movement, which are more often than not caused by completely different mechanisms [7]-[12]. Long-distance movement, although difficult to detect, is thought to occur often, and is fundamental to the rate and extent of spread [3], [9]. However, knowledge of the occurrence and of the mechanism of long-distance movement is often retrospective and assumed [13]. For example, there are several studies where the quantitative predicted rate of spread from diffusion models has been much lower than that observed [14], [15] or where transport by humans or wind is the most parsimonious explanation for rapid range expansion [7], [16], [17].

Absence of information on mode of movement, and especially long-distance events, is due in part to the impractical nature of collecting data on small animals like arthropods, and in part the extremely common single-scale approach of monitoring initial spread. A typical approach to understand dispersal of arthropods is a centre point release of the agent with capture stations radiating away from the release point up to some predetermined distance [5]. In the vast majority of cases, only a single scale is used. However, it is widely recognized that the mechanisms driving post-establishment patterns may be strongly influenced by spatial scale [18], [19]. Therefore, a consequence of working at small or single spatial scales is that larger-scale or multiple-scale patterns and processes can be overlooked or misinterpreted [20]. This is nowhere more evident than when trying to understanding how individuals move and populations spread, particularly the initial dynamics of spread [21].

The intentional introductions of biological control agents offer an excellent opportunity to monitor the initial movement and spread of invasive arthropods [22]-[24]. In turn, framing biological control introductions in an ecological invasion context can make contributions to the practice of biological control [25]. Parasitic hymenoptera are a group of mostly vagile, minute insects, critical for biological pest control [26], but their movement is still poorly understood and most likely underestimated [27], [28]. Of the 47 studies published since 1987 on parasitoid dispersal, only five measure dispersal on multiple spatial scales, and none within a single generation [29]-[33]. Here, using the intentional release of a geographically novel biological control agent (a parasitic hymenoptera, *Eretmocerus hayati* Zolnerowich & Rose (Hymenoptera: Aphelinidae)) we studied its initial dispersal and spread at three different spatial scales: first, the ‘local scale’, which is of the order of tens of metres around the release point, second, the ‘field scale’, hundreds of metres around the release point, and third, the ‘landscape scale’, kilometres around the release point. Further, we evaluated the proximate causes influencing the spread of *E. hayati*, including weather and their relationship with their host - the nymphal stage of *Bemisia tabaci* (Gennadius) (Hemiptera: Aleyrodidae) [4]. Finally, we fit models to spread data to identify patterns at each spatial scale. We show that an examination of dispersal at only a single scale would lead us to underestimate their initial dispersal, spread, and dominant dispersal mechanism.

## Methods

### Natural History

The parasitic wasp, *E. hayati,* parasitise its whitefly host, *B. tabaci,* by laying a single egg under the juveniles. The 1^st^–3^rd^ juvenile host instars are preferred. The wasp egg hatches and the 1^st^ instar burrows through the whitefly’s cuticle to become an endoparasitoid. The parasitoid develops within the whitefly juvenile, eventually killing it, and emerges from the 4^th^ instar as a winged adult. *Eretmocerus hayati* is <1 mm in size, and haplo-diploid with females produced from fertilized eggs and males from unfertilized eggs [34]. It is synovogenic (mature eggs as life progresses), lives on average 19 days with access to sugars, produces an average of 200 progeny and at 25°C completes a generation in 20 days [35].

*Bemisia tabaci*, a sap feeding insect, is a cryptic species complex composed of at least 24 species [36]. One member of the complex, Middle East – Asia Minor 1 (commonly known as either the B biotype or silverleaf whitefly (hereafter SLW)) has spread globally via trade in ornamental plants [37]. It was first detected in Australia in 1994 [38] and has since become an economic problem primarily in Queensland and to a lesser extent in coastal northern New South Wales and Carnarvon in Western Australia. It has four instars and a winged adult stage, and is haplo-diploid. Parasitoids of the genus *Eretmocerus* have proved particularly effective at controlling SLW, and in 2004, after non-target assessment, the authors obtained permission and permits from AQIS (Australian Quarantine Inspection Service) for the release of *Eretmocerus hayati* into Australia as a means to control SLW [39]. A detailed account of the source region, initial import and rearing can be found here [39].

The field studies detailed below did not involve protected or endangered species. All releases were conducted on vegetable growers’ properties as indicated in the release permits and funding agreement. No additional permits were required for the release at the location specified.

### Release and Post-release Sampling

**Figure 1:**
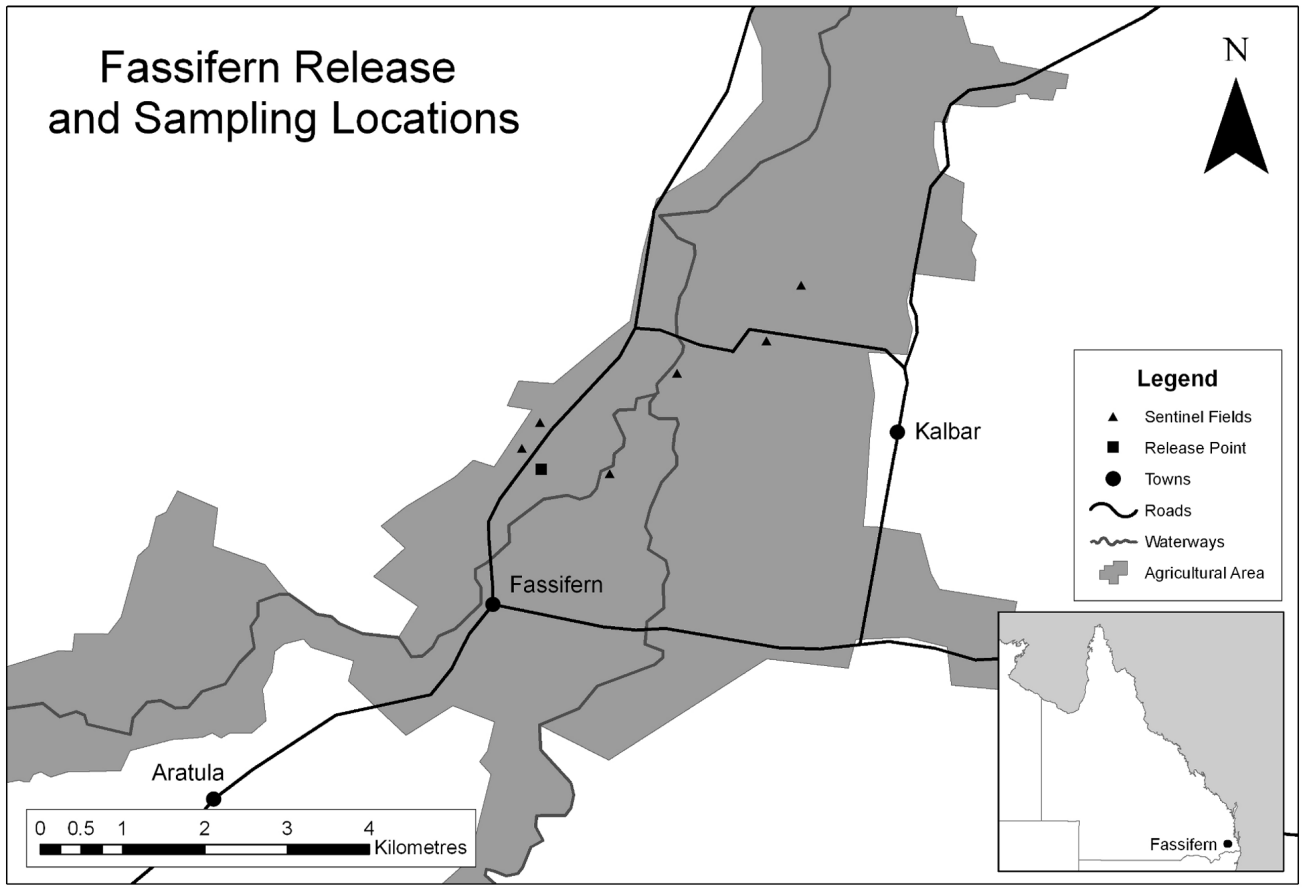
The ‘Kalbar’ site is situated in south-eastern Queensland, Australia, near the township of Kalbar and Fassifern. The sentinel collection fields were located at increasing distances from the release field in a north-easterly direction. There were no other sentinel fields within 5 kms of the release field.

A centre-point release of *E. hayati* was established at Kalbar, Queensland (27°56’47” S, 152° 35′04″E) in a 17 ha field of green beans (variety Yates Stringless Pioneer) at 8∶30 am on 12 March 2005, day 0 PR (Fig. 1). Four mesh bags measuring 35×15×10 cm were filled with soybean leaves infested with SLW pupae parasitised by *E. hayati.* Each bag was placed in a tray measuring 50×25×6 cm lifted off the ground by 8 cm, and lined up next to one another. The closest post-release sampling point was 2 m from the edge of the release cages in all cardinal directions, which translates to an undisturbed, unsampled area measuring 5×5 m. Over a period of three days approximately130,000 wasps emerged. This was verified by first viewing a subset of leaves in the field checking for the wasp-specific circular emergence hole in the exuvia. On the morning of 15 March, the third day PR, emergence bags were covered and removed from the field, and returned to the lab to confirm emergence. In the glasshouse prior to release, soybean plants infested with 1 st and 2 nd instar SLW nymphs were placed in *E. hayati* infestation cages for 4–6 hours, then removed and allowed to develop. Therefore the emergence window was narrow, and more than 98% of individuals emerge within the three day field release, and more than 80% within the first 36 hours (unpublished data).Weather data was collected from 24∶00 on 12 March using a Vantage Pro2 from Davis Instruments. Recordings included temperature, humidity, precipitation, wind speed and direction. Wind speed was averaged over the half-hourly interval. Wind direction was given in one of 16 compass directions.

A hierarchical sampling design was used to monitor the dispersal of *E. hayati* adults from the release point at three spatial scales, local (tens of metres), field (hundreds of metres) and landscape (kilometres), and is detailed in Table 1. In addition, Table 2 lists the crops and locations of each field used for monitoring relative to the release field. Dispersal was measured in two ways, first by carefully turning over leaves and counting any released adult *E. hayati,* and second by later removing leaves with silverleaf whitefly pupae and rearing out the first filial (F1 s) *E. hayati* generation in the laboratory. There is potential to disturb adult *E. hayati* during leaf turning, however, this method was evaluated prior to initiating the release. Disturbance was minimal, thus deemed to be most suitable. Leaflets collected for rearing were placed in containers to allow for the emergence of SLW and parasitoids. The emergence containers were checked every 48 hours and any emerged wasps were placed in a vial with 70% ETOH for later molecular analysis. DNA analysis was used, and verified that female *Eretmocerus* were the *E. hayati* species. Females of *E. mundus*, a species that is known to attack *B. tabaci* at very low levels [40], look similar to females of *E. hayati*. There is no confusion between males of the two species because *E. mundus* only produces females [41]. Microscopy data was used for the spatial and statistical analysis.

**Table 1:** A description of the sampling methodology for *E. hayati* adults and first filial generation at three spatial scales.

	Local Scale	Field Scale	Landscape Scale
**Count sampling dates**	15, 18, 21 March 2005 (3, 6, 9 days PR)	15, 18, 21 March 2005 (3, 6, 9 days PR)	22–23 March 2005 (10–11 days PR)
**Count sampling description**	Beginning 2 m from the release point,which translates to a 5×5 mundisturbed area,120 randomly chosen leaflets within a 4 mrow were turned over and adult *E. hayati*counted. This was repeated in each cardinaldirection and for each subsequent 2 m sample, until three consecutivezeros at 2 m distancesmoving away from the releasewere recorded.	An 83 point sampling grid, organizedin x, y coordinates, was established to cover the 17 ha bean field. Each pointspaced 50×50 m apart. Point 300, 200 was bare ground(a water pivot turning point), hence excludedfrom the grid. At 53 points 90 leaflets were turned over in a 1×1 m section. At the remaining 29 points 270 leaflets wereturned over in a 3×3 m section, for a total of 37,800 leafletsviewed for three sampling dates.	Leaflets were turned over to search for adult parasitoid for one person hour in each sentinel field in the landscape
**Leaf removal dates**	None	31 March 2005 (19 days PR)	31 March 2005 (19 days PR)
**Number and location of removed leaves**	None	5670 leaflets collected: 18 leafletsfrom each of the 53 1×1 m sections, and162 leaflets from 29 3×3 m sections	270 leaflets with at least a single SLW nymph present were collected from each sentinel field
**Emergence from removed leaves**	None	Leaflets from 22 points	All leaflets collected
**Microscopy of removed leaves**	None	Leaflets from 60 points were viewedwith the aid of a microscope to count3^rd^ and 4^th^ instar SLW nymphs anddetermine the number parasitised by *Eretmocerus* spp.	None

**Table 2:** Coordinates of sentinel plots, m = metres, used for monitoring dispersal on the landscape scale.

Field Name	x-coor. (m)	y-coor. (m)	Description	Plant stage at time of release
Release	0	0	Green beans	V1–V3, R3
300 NW	−175	300	Green beans	V2–V3
700 N	−75	675	Soybeans	V2–V3
700 E	700	0	Green beans	V2–V3
2000 NE	1500	1375	Green beans	R1
2900 NE	2375	1675	Green beans	V3
3500 NE	2750	2375	Green beans	V2

The release field is taken as (0,0), North as positive *y* and East as positive *x*. Fields are named by their radial distance and compass direction from the release field. Plant stages starting with ‘V’ indicate vegetative and ‘R’ reproductive.

### Spatial and Statistical Analysis

To understand some of the proximate causes influencing the spread of *E. hayati*, for example whether they aggregate to hosts, several relationships were evaluated including the: 1. spatial pattern (eg. aggregated vs random) of SLW nymphs, and of parasitized nymphs (the F1 generation of released *E. hayati*), 2. spatial association between the released *E. hayati* adults and parasitized nymphs, and 3. numerical relationship between nymph density and parasitized nymphs. To determine if nymph counts and parasitized nymph counts varied spatially from random we used the Spatial Analysis by Distance Indices (SADIE) method [42]. SADIE was developed for ecological count data in the form of spatially referenced counts. The basis is to quantify the spatial pattern in a sampled population by measuring the total effort (*D)* that the individuals in the observed sample must expend to move to an extreme arrangement, e.g., uniform or aggregated. Details about the indices can be found in [42] and [43]. Two populations may also be spatially associated, dissociated or random with respect to one another [44]. Spatial association was measured using the clustering index *X_k_*, which is based on similarity between the clustering index of the two populations, eg. released adult *E. hayati* and parasitized nymphs. The calculations to determine spatial association are equivalent to correlation coefficients. Details about the indices and significance tests can be found in [44]. The spatial pattern analyses and numerical relationships investigated are summarized in Table 3. To take advantage of all 60 microscopy points (those with both 162 and 18 leaves harvested) only the first 18 leaves of each of the samples were used in each of the analyses. Of the 22 remaining points evaluated for emergence, 13 of them had 18 leaflets, and so those 13 were included in the analysis of the spatial pattern of SLW parasitised and unparasitised hosts. All 22 samples were included in the analysis of the association between the *E. hayati* adults and parasitised nymphs because more sampling effort at some points should have no influence on association of two species. A logistic regression was used to examine the relationship between *E. hayati* parasitized hosts and host density. Three of the 60 points were outliers and dropped from the analysis. The 57 remaining points were spread across the 17 Ha plot.

**Table 3:** The relationships investigated, analysis method, and data used to test for spatial pattern, spatial association, and density dependent parasitism.

Relationship investigated	Statistical method	Microscopy data used	Emergence data used
Spatial pattern of SLW parasitised andunparasitised hosts	SADIE	60 microscopy points	13 container samples
Spatial association between the released*E. hayati* adults and parasitised nymphs	SADIE	60 microscopy points	22 container samples
Relationship between *E. hayati* parasitisedhosts and host density	Logistic regression	60 microscopy points, 3 outliers dropped	
Relationship between *E. hayati* and host densitywhen parasitism >0 at a sampling point	Logistic regression	21 microscopy points, excluding points without hosts or parasitism	

## Results

### Assessing Spread: Released Adult Parasitoids

#### Local Scale

**Figure 2:**
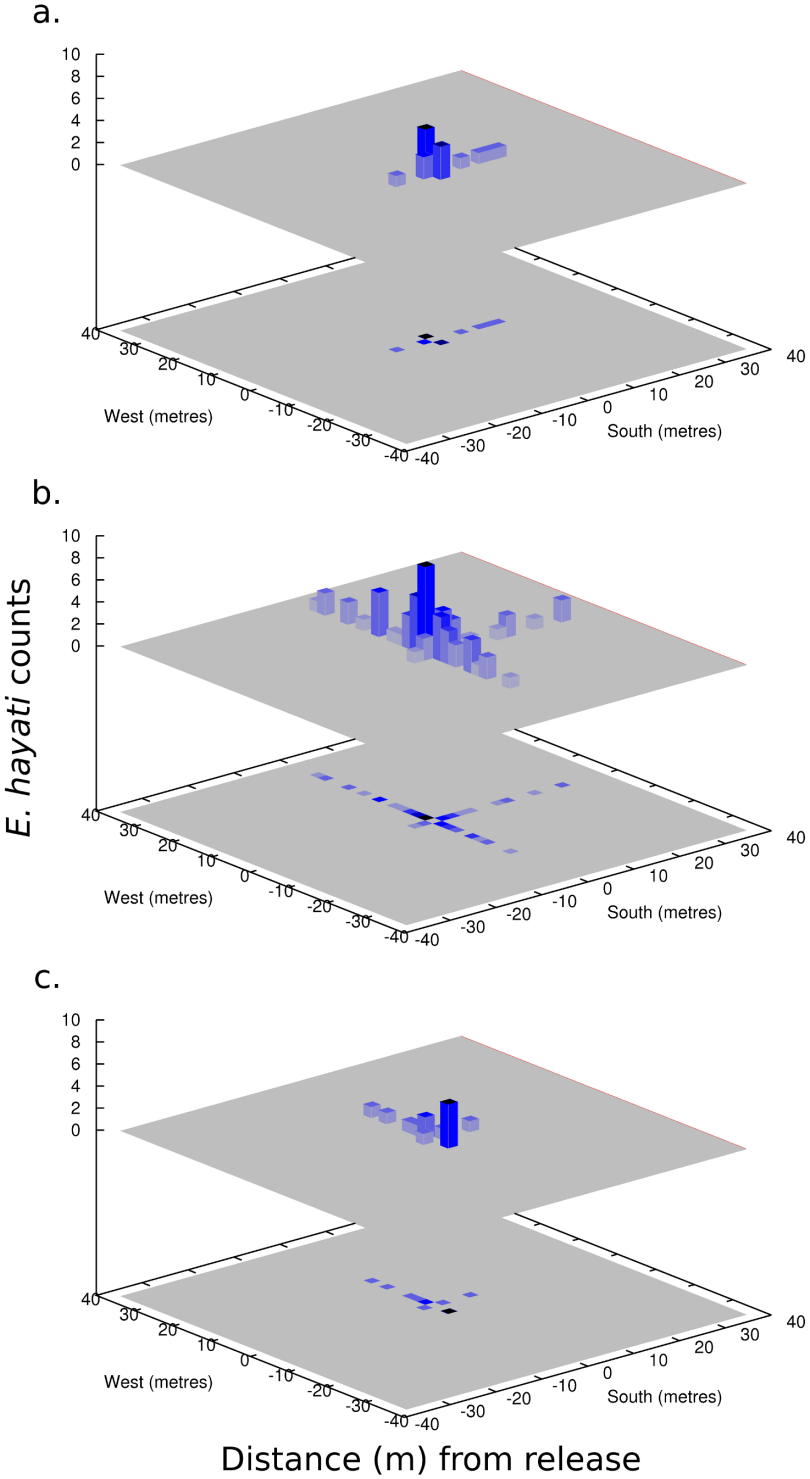
*E. hayati* adult counts around the release point (a) 3, (b) 6, and (c) 9 days post-release.

Three days PR, a large number of *E. hayati* were observed near the release cages (personal observation). Based on the post-release monitoring, a small number had ventured out to a maximum distance of 14 m (Fig. 2a). By six days PR, *E. hayati* had spread considerably to a maximum of 32 m, with a slight preference for the north and east direction (Fig. 2b). By nine days PR, *E. hayati* had declined, but the preference for north and east dispersal remained (Fig. 2c). The mean daily temperature recorded over the period was fairly constant, ranging from 19 to 24°C, with very little precipitation; 0.25 mm and 0.51 mm on 14 and 28 March, respectively.

#### Field Scale

**Figure 3:**
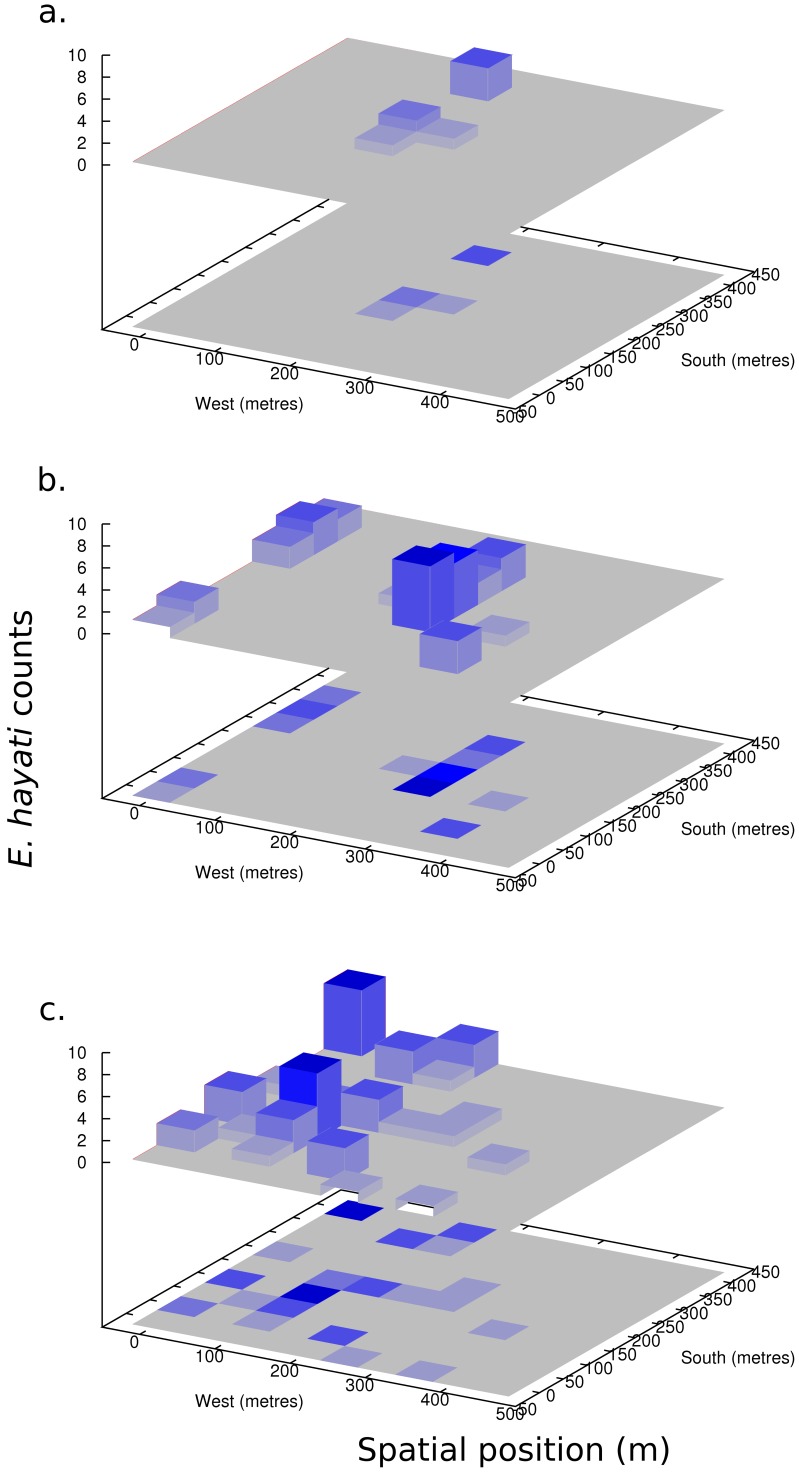
*E. hayati* adult counts in the 17 Ha field (a) 3, (b) 6, and (c) 9 days post-release. Values shown are number of individuals weighted by the number of leaves searched at each grid-point resulting in a density of seven, 31, and 44, adults, respectivley.

The total number of *E. hayati* found at 3, 6 and 9 days PR in the 17 ha release field, excluding the local scale counts from above, was 5, 21, and 24 individuals, respectively. Three days PR, most of the *E. hayati* counted were found around the centre of the field, with the closest grid point being 25 m from the release cages (Fig. 3a). By six days PR, the spatial pattern had become bi-modal, with a high concentration of individuals in the centre, and a high concentration in the northern edge of the field (Fig. 3b). The counts in the north- northwesterly direction coincides with the wind-run (total distance of traveled wind over a period of time), and wind direction, which mostly came from the southeast from 12 to 18 March 0–6 days PR. By nine days PR, the bi-modal shape was gone, and instead the population had spread and shifted more strongly to the northwest edge of the field (Fig. 3c). For the two days prior to this count, there had been a very strong wind-run from the southeast with speeds frequently over 2 m/s in the mornings. There were a few days in which some wind came from a northerly direction later in the day. The first time that this occurred, was on 16 March (3 days PR). Subsequent days with a northerly wind component were 17, 22–25, and 29 March. Generally, during the study, morning speeds tended to be lower than those later in the day.

### Assessing Spread: the F_1_ Generation

#### Field Scale

**Figure 4:**
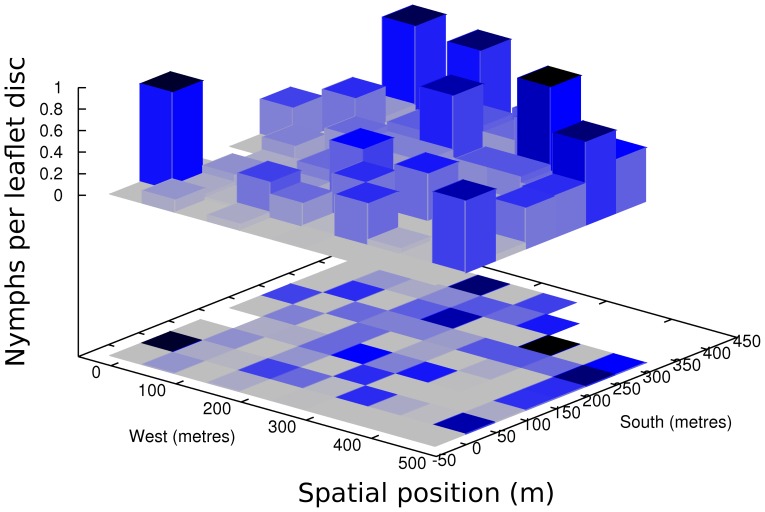
Emergence of whitefly from leaves sampled from release Field 0. The y axis is counts of nymphs per leaflet disc collected on 31 March 2005.

A male *E. hayati* was the first F1 to emerge on 3 April 2005 (21 days PR) from a sampling point 25 m from the release. The majority (64%) of male and female *E. hayati* emerged on 5 April (23 days PR). However, individuals continued to emerge up to 41 days PR, which is most likely due to a proportion of *E. hayati* females staying in the field. *Eretmocerus hayati* host feed and feed on honeydew of whitefly, which has been shown in other *Eretmocerus* spp. to result in 50% of the population alive 5 days post-emergence [45]. The within field spatial pattern of parasitized nymphs was random (*I_a_* = 0.869, *P_a_* = 0.7831, *v_j_* =  −0.843, *P_J_* = 0.8577, *v_i_* = 0.849, *P_i_* = 0.8498; *I_a_* >1.5, *P_a_* <0.025, two-tail distribution). *I_a_*>1.5, *P_a_*<0.025 would indicated significant spatial aggregation [44]. The data represents many days of *E. hayati* attacking nymphs, hence daily spread is indistinguishable. There was no spatial association between *E. hayati* released adults and parasitized nymphs (*X* = 0.1128, *P* = 0.1678; *P*<0.05 for positive association). There was a spatial pattern other than random of SLW nymphs, they were aggregated into patch and gap areas (*I_a_* = 1.613, *P_a_* = 0.0044, *v_i_* = 1.569, *P_i_* = 0.0044, *v_j_* =  −1.591, *P_j_* = 0.0042). The patches were primarily along the east and south (Fig. 4), not the area where *E. hayati* adults were found.

There was no relationship between proportion parasitized nymphs (the *F_1_* generation) and nymph density (*n* = 57, *X^2^_1,56_* = 1.10, *P* = 0.295, β = −0.1014). However, an analysis only considering points in the field where parasitism was >0, (confirmation that the parasitoid located the host) showed there was a significant negative relationship between parasitized nymphs and nymph density (*n* = 21, *X^2^* = 9.43, *P* = <0.0021, β = −0.2914).

#### Landscape Scale

**Figure 5:**
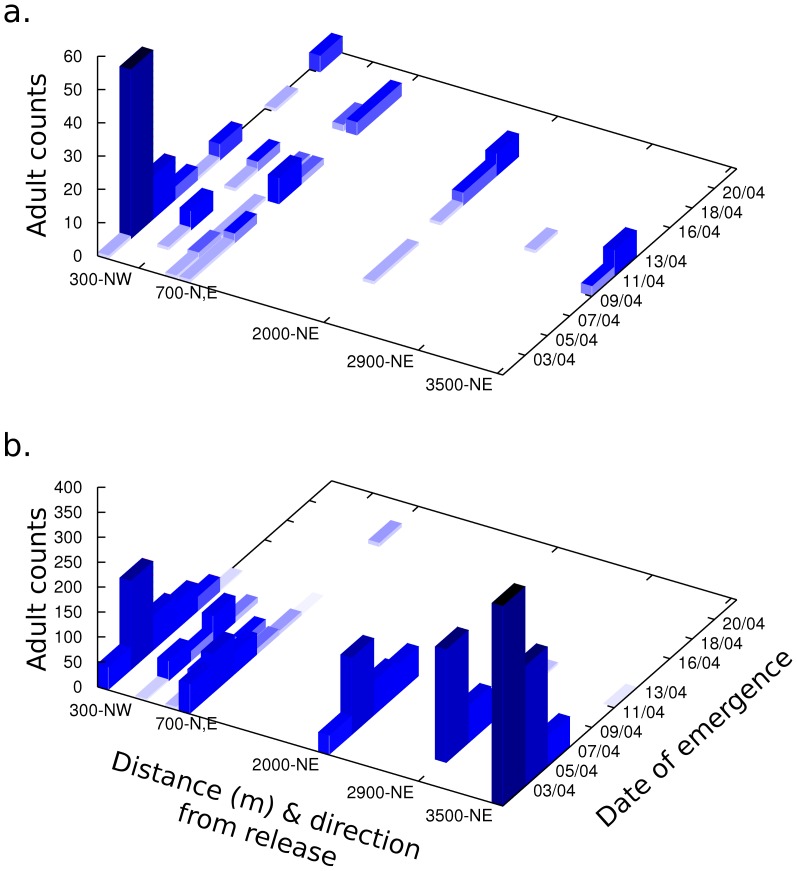
Emergence of adult (a) *Eretmocerus hayati* and (b) SLW from leaves sampled from Field 0 and each of the five sentinel fields.

The further the distance of the sentinel fields from the release field, the later the emergence of *E. hayati* (Fig. 5a). On 3 April, two females emerged, one each from a sentinel field 300 m and 700 m from the release field. Four days later the first female emerged from a field 2000 m away. Eight days from the first emergence, three females emerged from the furthest sentinel field 3500 m away. Emergence of SLW spans one week (Fig. 5b), with the exception of one individual, confirming that later emergence of parasitoids from distant fields was not due to differences in SLW emergence times.

### The Necessity of Hierarchical Sampling Design for Inferring the Dominant Dispersal Mechanisms

The previous sections describe observational data on three different scales which, on their own, give quite different information about the dispersal of *E. hayati*. In this section, the question is asked: what would one hypothesize about the dominant dispersal mechanism assuming that one was not aware of the dispersal data of other spatial scales? To answer the question, we use a mix of qualitative and quantitative model fitting, aimed at producing model predictions that capture the main qualitative features of the count and emergence data. Each of the three models represents interpretation that one would likely make of single scale data.

#### Modelling: local scale

Taken alone, the count results of Fig. 2 suggest that *E. hayati* will confine their dispersal to the local scale, tens of metres from the release point. This result is comparable to Simmons [46], who reported that 95% of *E. eremicus* travelled 14.8 m or less over 8 days, and who successfully fitted a Gaussian redistribution kernel to the data. A likely method to employ, but perhaps some modellers would use instead a biased redistribution kernel to account for the east-west bias that is likely caused by the species' phototactic response to the rising and setting of the sun [47]-[49].

**Figure 6:**
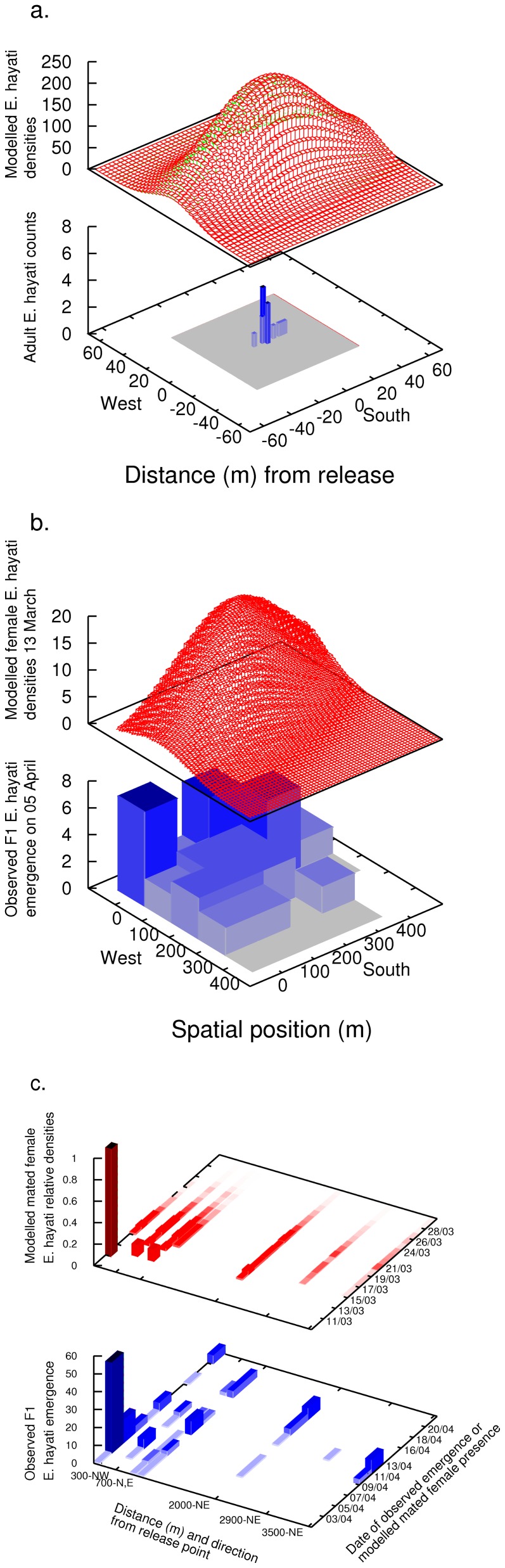
The observation scales and three corresponding models of parasitoid dispersal. On the local scale (a), dispersal is modelled by east-west diffusion; on the field scale (b) by a combination of wind-advection and diffusion; and on the landscape scale (c) by wind-advection only.

Brewster [50] provide such a kernel and a first estimate for its parameter values,
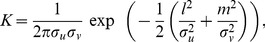
(1)where *l* and *m* are east-west and north-south positions, and and are the standard deviations of dispersal in the east-west and north-south directions. The redistribution kernel can be interpreted as the probability distribution function of the location of an individual after the specified period of time (in this case, daily) that was originally located at (*l,m*) = (0,0). For a population of individuals in various locations, the new positions are found by summing over all individuals, which may be computed efficiently with the use of fast fourier transforms. Works by Brewster and Allen [50] and Brewster [51] provide an introduction to this technique in the context of *Eretmocerus* and *Bemisia* dispersal modelling. Table 4 gives a range of values fitted for whitefly dispersal, depending upon the host plant. *Eretmocerus* spp. was assumed by them to have a dispersal of twice that of the whitefly. East-West dispersal ranged from 10 sq-m day^−1^ to 32.4 sq-m day^−1^, and North-South dispersal ranges from 5 sq-m day^−1^ to 16.2 sq-m day^−1^. Taking the median of values from [51] of *σ_u_*  = 21.2 sq-m day^−1^ and *σ_v_*  = 10.6 sq-m day^−1^ results in Fig. 6a, which shows reasonable qualitative agreement given the resolution of the data, and compares well with the literature. This suggests that if one only had access to the local scale data, *E. hayati* dispersal could reasonably be hypothesised to be purely local, random, and augmented only by its phototactic response.

**Table 4:** Parameters and values used in models on the three spatial scales.

Parameter	Meaning	Value of range	Justification
Local Scale			
σ*_u_*, σ*_v_*	Standard deviation of dispersal east-west, north-south	21.1 sq-m day^−1^_,_ 10.6 sq-m day^−1^	Median value taken from [50] with a phototactic response to the rising and the setting of the sun
Field Scale			
σ*_u_*, σ*_v_*	Standard deviation of dispersal east-west, north-south	211 sq-m day^−1^_,_ 106 sq-m day^−1^	Ten times local scale value for qualitative fit
u,v	Wind-shift distance east-west, north-south	104–1088 m day^−1^	Found by Equation 3 with parameters *f, w, L* fitted by genetic algorithm from the landscape scale (below)
Landscape Scale			
L	Length of time mated females fly	10.2 min day^−1^	[53]
f	Dimensionless scaling factor	1	Fitted by genetic algorithm
w_u_, w_v_	Half-hourly mean wind speed east-westnorth-south	0.2–1.8 m s^−1^	Recorded at release site, but only applied when between 6.30 am and 5∶00 pm and <2.2 m s^−1^ as fitted by genetic algorithm

#### Modelling: field scale

The results at the field scale (hundreds of metres), in both the leaf turn counts (Fig. 3) and observed emergence (Fig. 6b), suggest a different mode of *E. hayati* dispersal than the results on the local scale (Fig. 2). *E. hayati* disperse at least hundreds of metres, and with a north-westerly bias coinciding with the wind run. A modeller presented with this data would likely employ a Gaussian redistribution kernel similar to Equation 1, but with a wind-advection component to account for the female *E. hayati* wind-assisted dispersal, and fit a higher standard deviation of dispersal to account for the larger spread.

Advection may be included in Equation 1 by introducting a wind-shifted east-west (*u*) and north-south (*v*) positions as follows.
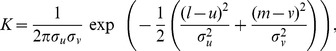
(2)


The wind-shift components may be estimated by
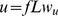


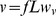
where *f* is a dimensionless scaling factor (which accounts for error in the mean flight time, and the fact that the insects will not fly at the exact same speed as the wind), *L* is the average flight time, and *w_u_* and *w_v_* are the east-west and north-south wind speed components respectively. Parameter values and their justifications can be found in Table 4, with details of their derivation available in the Supplementary Information (File S1).

In contrast to the model on the local scale, this field scale model describes *E. hayati* dispersal as a combination of random movement augmented by phototactic response, but with an additional wind-assisted component. It describes a greater dispersal capability than [46], however it is still consistent with the literature regarding the phototactic response [47], [48] and the potential influence of the wind [48]. The procedure used here also illustrates another reason why sampling on multiple scales can be helpful. Even if the field scale was the only scale of interest, data and model fitting from the landscape scale was necessary to parameterise the field scale model. So information from sampling on larger scales can be used to explain smaller scale behaviour.

#### Modelling: landscape scale

The emergence data of the F1 *E. hayati* on the landscape scale demonstrates that mated females were able to disperse several kilometres from the release point, suggesting a wind-borne dispersal mechanism. The data is also necessarily sparser than on smaller scales, and consequently cannot provide information about the diffusion-component of parasitoid dispersal. A modeller presented with this data would most likely attempt to fit a simple wind-advection model, where the dispersal of females was in the direction of the wind vector and proportional to the wind speed [52]. This reasoning leads to a description of females’ position *p*(*t+1*) at day *t+1* as
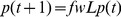
(3)


Parameter values and their justifications can be found in Table 4, with details of their derivation available in the Supplementary Information (File S1). A sample comparison between the performance of this fitted wind-advection-only model and the observed emergence data on the landscape scale is shown in Fig. 6c. In contrast to the local scale model, the landscape scale model emphasises the role of wind-borne dispersal and identifies likely conditions and constraints influencing this dispersal mode.

## Discussion

*Eretmocerus hayati* exhibits stratified dispersal, moving further, faster and by a different mechanism than would have been concluded with a single local-scale post-release sampling design. Our design, in-line with aspects of the conceptual framework for movement proposed by [4], allowed us to explore several causes, mechanisms, and patterns of *E. hayati* movement. If the local-scale adult parasitoid count had been the only data collected, then from the shape of the distribution, one may have concluded that the data collection had captured the extent of the dispersal. Confidence in this conclusion would have also been bolstered by the literature on related species *E. eremicus* (e.g. [46], [51]) for which dispersal has been observed confined to the field scale (tens or hundreds of metres), and modelled using diffusion processes. Similarly, the study by Simmons [46] reported that 95% of *E. eremicus* travelled 14.8 m or less over 8 days. Again, a Gaussian dispersal kernel was fitted to the data, assuming that *Eretmocerus* disperse mainly at random, and not much further than hundreds of metres. Only study [53] show evidence of wind directed dispersal by female *E. eremicus*, but at a single small scale (10 m), and in habitats with few hosts. Instead, our observations on three spatial scales reveal that *E. hayati* disperses kilometres from the release point, and soon after release. The results for the release field show first generation parasitoid emergence occurred several hundred metres from the release point, and in the direction coinciding with the dominant wind direction, and not associated with the spatial distribution or density of their host. Therefore, we can rule out host density and distribution and complete self-direction of the parasitoid as the cause of subsequent parasitoid spread. However, we can not rule out density dependence as a cause of dispersal. *Eretmocerus hayati* emerging in-mass over a few days may experience interference, which may cause them to move faster or by different modes than would be expected under low density conditions; as demonstrated with herbivores [54]. Yet, there is no reason to suspect that stratified dispersal only happens at high density. The evidence for density-dependent dispersal for arthropods is mixed, with examples of the opposite [55], and recognition that other factors can contribute to leaving including host availability and parasitoid experience [56]. In the release field there was no indication that competition for hosts was high. The total parasitism rate for the field was 17% and the total parasitism from the closest points near the release (25 m away) was 18%. Further, *E. hayati* would often be in conditions of high density. In Australia, SLW density of 10 4^th^ instar nymphs/cm^2^ is not uncommon [39]. When considering the large leaves of cucurbit crops, a favourite host of SLW, 10 s of 1000 s of SLW nymphs and *E. hayati* can be present within a square metre. In turn, *E. hayati* can be found attacking SLW at low densities on isolated weeds [39]. Regardless, a single scale approach would have missed the stratified dispersal mode and rate of spread, and our results indicate that models that do not include both short distance (diffusion) and long-distance (wind-advection) dispersal are not suitable for *E. hayati*, and perhaps most flying arthropods. Further, the F1 emergence results for the sentinel fields revealed that this wind-assisted dispersal can transport *E. hayati* several kilometres from the release site in one generation.

Of the five studies measuring dispersal on multiple spatial scales, one inferred long-distance dispersal through analysis of population genetic structure [33], one measured dispersal at a monthly resolution over one year [29], and another measured long-distance dispersal over years [30]. The study by Chauzat [32] did monitor both within-field and sentinel fields for the duration of a *Psyllaephagus ilosus* biocontrol release, which had the potential to detect long-distance dispersal within a generation. However, dispersal to sentinel fields only occurred after their second generation, yet this was a delay attributed to adverse weather conditions. Another study measured dispersal of a single generation of *Trichogramma ostriniae* on multiple scales, but in two separate investigations [31]. Similar to the case with *Eretmocerus* spp., previous studies on *T. ostriniae* had not attempted to measure dispersal over large areas. The preliminary findings from [31] on multi-field investigation informed the spatial extent of the detailed follow-up study [31], which demonstrated much higher dispersal distances than previously reported or than would have been expected from that literature.

These results echo the observation by [57] that the maximum dispersal distances (less than 100 m) commonly reported for parasitoids do not correspond with dispersal observed across fragmented habitats. Low dispersal estimates may rather be an artifact of the restricted scale of these studies themselves. It has long been known that parasitoids undertake significant wind-borne dispersal, being found in air samples taken by planes or balloons thousands of metres high [58], [59].

Environmental heterogeneity is known to influence many stages of the invasion and spread process [3], [15], [60]. Different types of habitats, their spatial arrangement, and their suitability to the organism are all likely to influence dispersal, colonization and population growth. Our spread results are based on a single landscape. These results may be quite different in a landscape with different availability and spatial arrangement of host plants, and hosts. However, in a parallel paper [52], we developed a model based on the results from this study, and tested the predictions with a release of *E. hayati* in a novel environment containing a very different release plot, spatial arrangement of host patches and different types of habitats. The model fitted fairly well. This is promising because the difficulty with including environmental heterogeneity in models is complex, and models tailored for specific environmental details limit general conclusions [15].

What are the implications of these results for stopping the spread of unintentional invasions or accelerating the spread of biological control agents? Liebhold and Tobin [3] provide a detailed account of management activities that practitioners should consider to slow, stop or reverse spread. For each of these, defining the boundary of the area beyond which is free from the invader is a primary interest. Our results suggest that the boundary needs to be extended when organisms are able to use wind directed dispersal. Biological control practitioners are often faced with deciding on a release strategy that balances resources (e.g. number of agents to release) with impact (e.g. placing agents in as many places as possible as quickly as possible, [61]). Agent establishment will depend on an effective population size (avoiding Allee effects), and spread will depend on the coupling of dispersal with population growth [3]. Assuming that the agent establishes, our findings suggest that highly vagile organisms may move more quickly than anticipated by stratefied dispersal, but this will also depend on their species-specific biology and abundance of host/prey patches. For example, parasitoids living where hosts patches are abundant, allowing them to save energy and time finding hosts, may trade-off dispersal and longevity for a pro-ovigenic strategy (born with a fixed compliment of eggs [56], [62], [63]. *Eretmocerus* spp are autogenous and synovigenic (maturing eggs as life progresses) suggesting that dispersal is an important part of their life-history.

Even when it appears that the spread of an invader is confined within the area searched, for example, when the distribution is observed to have a characteristic tapering shape with tails confined well within the sampling area, this study shows that it is still possible for individuals to be found outside of that area. This can significantly change model parameters and interpretation of the invader's spread to one that moves further, faster, and by multiple mechanisms, which has obvious implications for both invasions and biological control. Consequently, the inclusion of sentinel field sampling around an intensive within-field sampling programme is a cheap and effective way to detect this component of dispersal, which has important implications for biocontrol and regional population dynamics.

## Supporting Information

File S1 Details of parameter value estimation.
(DOCX)
