# Supplementary material for: The Initial Dispersal and Spread of an Intentional Invader at Three Spatial Scales
Source: PLoS One. 2013 May 6;8(5):e62407. doi: 10.1371/journal.pone.0062407 (PMC3646002; doi:10.1371/journal.pone.0062407)
Supplement: File S1 — Details of parameter value estimation. (DOCX) [file pone.0062407.s001.docx]

# Supplementary Material (File S1)

## Details of parameter value estimation

### Modelling: field scale

The mean flight time of mated females observed in flight chamber experiments [52] may be used to give an estimate of *L=10.2* min day^-1^. The value of the dimensionless scaling factor *f* is determined by model fitting, along with the standard deviations of dispersal. Half-hourly wind speed and direction recorded at the site provides a starting point for *w* value estimates. However one must still determine: at which times of the day flight was undertaken, how to apportion the population to flights during each half-hourly interval of the day, and if there was an upper limit to wind speed at which females would fly, for example. This greatly increases the number of parameter values that must be fitted via the model to the data, and because of the coarseness of the data, exceeds the model's ability to identify a unique set.

In the landscape scale model, a set of parameter values and flight conditions were fitted using a genetic algorithm to data from the landscape scale (see Table 4 for values). If these parameters and conditions are imported into the field scale model, the *E. hayati* emergence in the north-west corner of the field corresponds to dispersal of mated females on 13 March (the date of first emergence or release *E. hayati*), from flights undertaken between 6.30 am to 8.00 am. This leaves only the standard deviations of dispersal to be fitted. The standard deviations of east-west and north-south dispersal were set to 10 times the values used on the local scale. This multiplicative factor was chosen somewhat arbitrarily, to provide enough qualitative agreement between the model and the emergence data for illustrative purposes (Fig. 6b).

### Modelling: landscape scale

The 8 × 8 km area surrounding the release point was divided into a grid with squares measuring 250 × 250 m each. A redistribution kernel for an advection model can be coded as a sparse matrix with entries corresponding to the proportion of females moving to each grid position during each half-hourly interval. Assuming a single wind-borne flight per day [52], the x,y grid position p of a given female at day t+1 can be calculated from a combination of Equation 3 and its position at day t, p(t)

$p(t+1)=fwLp(t)$ (3)

A value of *L=10.2* min day^-1^ can be estimated from the mean flight time of mated females observed in flight chamber experiments [53], which leaves the scaling factor *f* and the wind velocity to be fitted to the data. The fitting of the wind velocity involves determining how to convert the half-hourly wind velocity recorded at the site into the wind velocities that apply to the flights of the *E. hayati* females. It was found that the model was sensitive to two conditions for flight, and a genetic algorithm was used to determine their specifications: the hours of the day at which a female would undertake the flight, and the maximum wind speed at which females would safely undertake the flight. Full details may be found in a related paper [52], but a brief description is that a genetic algorithm found that the fit was optimised when: flights were only taken at wind speed under 2.2 m s^-1`^during daylight hours between 6.30 am and 5.00 pm each day, and a scaling factor of *f=1* was used. The model and parameter values were then validated against a later release of *E. hayati* in Carnarvon, Western Australia [52].
